# Individual Variability in Reproductive Success Determines Winners and Losers under Ocean Acidification: A Case Study with Sea Urchins

**DOI:** 10.1371/journal.pone.0053118

**Published:** 2012-12-27

**Authors:** Peter Schlegel, Jon N. Havenhand, Michael R. Gillings, Jane E. Williamson

**Affiliations:** 1 Department of Biological Sciences, Macquarie University, Sydney, Australia; 2 Department of Biological & Environmental Sciences, University of Gothenburg, Tjärnö Marine Biological Laboratory, Strömstad, Sweden; University of Gothenburg, Sweden

## Abstract

**Background:**

Climate change will lead to intense selection on many organisms, particularly during susceptible early life stages. To date, most studies on the likely biotic effects of climate change have focused on the mean responses of pooled groups of animals. Consequently, the extent to which inter-individual variation mediates different selection responses has not been tested. Investigating this variation is important, since some individuals may be preadapted to future climate scenarios.

**Methodology/Principal Findings:**

We examined the effect of CO_2_-induced pH changes (“ocean acidification”) in sperm swimming behaviour on the fertilization success of the Australasian sea urchin *Heliocidaris erythrogramma,* focusing on the responses of separate individuals and pairs. Acidification significantly decreased the proportion of motile sperm but had no effect on sperm swimming speed. Subsequent fertilization experiments showed strong inter-individual variation in responses to ocean acidification, ranging from a 44% decrease to a 14% increase in fertilization success. This was partly explained by the significant relationship between decreases in percent sperm motility and fertilization success at ΔpH = 0.3, but not at ΔpH = 0.5.

**Conclusions and Significance:**

The effects of ocean acidification on reproductive success varied markedly between individuals. Our results suggest that some individuals will exhibit enhanced fertilization success in acidified oceans, supporting the concept of ‘winners’ and ‘losers’ of climate change at an individual level. If these differences are heritable it is likely that ocean acidification will lead to selection against susceptible phenotypes as well as to rapid fixation of alleles that allow reproduction under more acidic conditions. This selection may ameliorate the biotic effects of climate change if taxa have sufficient extant genetic variation upon which selection can act.

## Introduction

Environmental factors directly affect populations by selecting resilient individuals. Selection at the gametic level, or during early life, has strong and immediate effects at the population level, carrying over into subsequent life stages. Heritability of this resilience leads to cascading adaptive effects in subsequent generations. For example, in free-spawning marine organisms, sperm selection during fertilization plays a key role by determining the nature and diversity of genotypes in the subsequent generation [1], [2] and thus their resilience to environmental change.

Rising atmospheric carbon dioxide levels are a key driver of environmental change, and will likely lead to rapid ocean acidification [3], [4]. With gametes possessing no, or only limited, buffering capacities against CO_2_-mediated pH changes in seawater, the dynamics of fertilization and subsequent development are likely to be affected in all free-spawning marine organisms, with potentially severe implications [5], [6]. Yet we know little about the relative fitness of individuals within species under the predicted acidification of the ocean.

The sensitivity of reproductive processes to ocean acidification has thus far been assessed from mean responses of mixtures of gametes and/or larvae obtained from multiple individuals [7]–[10] (but see [11]). However, the key determinant of reproductive success in a future ocean is not the average response, but the proportion of successful offspring contributed by each individual under the changed environmental conditions. Individual-level responses to ocean acidification have been examined to some extent in larval development processes [12], [13], but not closely in fertilization processes. In this context, the importance of naturally high variability that is observed in fertilization success of individual pairwise crosses [14], [15] becomes apparent: not all matings are equal. Consequently, acidification-mediated impacts on reproductive success and subsequent development might result in flow-on consequences for genetic diversity and population demographics [12].

The early life history stages of echinoderms are particularly useful for studies of fertilization success, as these species are experimentally tractable and ecologically important, often acting as ecosystem engineers [16], [17]. Here, we investigate the effects of CO_2_-induced ocean acidification on the early life history stages in the Australasian sea urchin *Heliocidaris erythrogramma,* focussing on intra-specific variation in responses, which can be highly variable for this species [18]. Following the A1FI-scenario from the IPCC’s 4^th^ assessment report [3], we compared the effects of present day conditions for southeast Australia with the end-of-century scenario (year 2100; *p*CO_2_ = 970 µatm≃0.3 pH unit reduction) and a high-CO_2_ scenario (year 2300; *p*CO_2_ = 1600 µatm≃0.5 pH unit reduction). Observed effects on sperm swimming behaviour were applied within an established fertilization kinetics modelling framework [19], [20] to predict fertilization outcomes of single urchin pairs at each *p*CO_2_ level. These were then compared to observed results from fertilization experiments conducted in the laboratory.

## Materials and Methods

### CO2 Treatment

Experimental CO_2_ treatments were achieved by bubbling a mixture of air and CO_2_ through filtered seawater (FSW; 0.22 µm filtered). pH (NBS scale) was maintained using microprocessor-controlled CO_2_ micro-injection systems in separate FSW-tanks. Systems were set to maintain a pH change (ΔpH) of −0.5, −0.3 or 0 pH units (control, no addition of CO_2_), resulting in treatment pH values of 7.6, 7.8 and 8.1. Dissolved oxygen levels were maintained by slow bubbling of filtered air. pH levels were checked prior to experiments. Total alkalinity was determined once by titration [21] (all seawater used in experiments was taken from a closed recirculating system in which alkalinity was controlled to be constant). Parameters of the CO_2_ system (Table 1) were calculated with CO2SYS [22] using the dissociation constants of Dickson & Millero [23].

**Table 1:** Seawater parameters for the three different pH treatments.

pH_NBS_	T (°C)	Sal	A_T_ (µeq kg^−1^)	*p*CO_2_ (µatm)	Ω_Ca_	Ω_Ar_
8.12±0.06	20.5±1	35.38±0.06	2073±4	413	3.674	2.392
7.80±0.01	20.5±1	35.38±0.06	2073±4	952	1.949	1.269
7.60±0.01	20.5±1	35.38±0.06	2073±4	1572	1.277	0.831

pH**_NBS_**, temperature (T), salinity (Sal) and total alkalinity (A_T_) were measured directly and used to compute partial pressure levels of carbon dioxide (*p*CO_2_) and seawater saturation states for calcite and aragonite (Ω_Ca_ and Ω_Ar_ respectively) using CO2SYS. Means ± S.E.

### Experimental Animals

*Heliocidaris erythrogramma* (test diameter = 50.3±1.3 mm, mean ± S.E.) were collected during their spawning season between February and March 2011, from shallow subtidal areas at Long Bay (33°57.5′S, 151°15.2′E) and Bare Island (33°59.2′S, 151°13.5′E), Sydney, Australia. Animals were immediately transported to Macquarie University and held in tanks with flowing seawater for up to one week maximum before being used for experiments. Individuals were collected fresh each week, alternating between sites and those from different sites were never mixed. Three collections were done at Long Bay (males/pairs A–D on 7 Feb 2011, J–M on 21 Feb 2011, R and S on 14 March 2011; Table 2) and two at Bare Island (males/pairs E–I on 14 Feb 2011 and N–Q on 1 March 2011; Table 2).

**Table 2:** Ocean acidification effects on sperm speed and percent sperm motility for each male *Heliocidaris erythogramma*.

		Sperm speed (µm s^−1^)			Percent motility (%)		
pH		7.6	7.8	8.1	7.6	7.8	8.1
Male	Site						
A	1	36.19	33.80	33.64	23.12	20.88	23.29
B	1	37.67	36.72	36.48	36.62	35.94	46.27
C	1	37.11	35.72	36.47	45.13	48.34	56.16
D	1	37.79	39.11	38.40	53.24	54.55	54.61
E	2	35.91	37.16	38.27	26.91	22.38	32.45
F	2	42.85	42.12	42.87	51.21	43.08	57.13
G	2	37.32	38.06	38.27	36.41	38.35	51.37
H	2	44.75	44.99	44.38	43.70	45.44	51.38
I	2	38.33	39.66	39.44	29.96	32.84	36.63
J	1	37.57	36.29	39.87	41.30	40.99	48.96
K	1	40.92	42.43	41.35	59.93	62.42	64.89
L	1	39.29	39.61	40.34	37.64	46.39	50.70
M	1	36.03	37.65	37.45	30.52	31.44	28.87
N	2	39.54	40.24	38.63	58.68	65.98	66.68
O	2	34.67	35.95	35.99	44.32	51.03	54.79
P	2	37.57	36.33	33.66	56.66	53.40	62.93
Q	2	41.18	41.32	39.19	53.23	60.35	57.81
R	1	35.76	34.14	34.64	70.00	68.86	74.13
S	1	41.88	41.54	38.40	45.40	53.60	56.64
**mean**		38.56	38.60	38.33	44.58	46.25	51.50
**S.E.**		0.32	0.32	0.36	1.08	1.17	1.10
***P*** ** between sites**		0.348	0.194	0.388	0.761	0.946	0.965

Significant differences in parameters between sites (*P*≤0.05) are shown in bold.

### Collection of Gametes

Gametes were obtained by injecting urchins with 0.8–1 ml of 0.5 M KCl through the peristomal membrane, followed by gentle shaking. This concentration of KCl reliably induced gamete release without being lethal. Individuals were used once (for convenience, the term “individual” will be used here to refer both to individual males [for sperm speed and motility measures] and to individual pairs [for fertilization success measures]). Eggs were collected in FSW, diluted to a concentration of 50 eggs ml^−1^ and incubated in seawater at one of the three pH conditions for 10 min prior to use in experiments. Sperm were collected “dry” and held on ice until use to extend their lifespan. Experiments were conducted immediately after spawning. All experiments were done in a constant temperature room at a standard temperature of 20.5±1°C (mean ± range).

### Sperm Motility and Speed

Data were obtained for each of 19 male urchins. The motility assay followed that of Havenhand & Schlegel [24]. Briefly, 0.5–1 µl of freshly collected sperm from an individual male was diluted into 1 ml of seawater of each pH immediately prior to use (10 replicate sperm suspensions for each CO_2_ treatment and male). Sperm concentrations across assays ranged consistently from 1–2*10^4^ sperm µl^−1^. A 60–70 µl drop of sperm suspension was placed between an albumin-coated microscope slide and coverslip, separated by a 0.75 mm thick O-ring. Sperm movement was recorded for 2s at the midpoint of the drop, at 25 frames s^−1^, using a digital camera (Sumix SMX-160) mounted on a compound microscope (Olympus BX51). Pilot experiments showed illumination by the microscope lamp has no impact on the temperature inside the drop during videotaping (time of slide on microscope: approx. 10s). All recordings (one per sperm suspension) were done within 30s after creation of each sperm suspension. Videos were post-processed and analysed using CellTrak 1.3 (Motion Analysis Corporation) to determine sperm speed and percentage of motile sperm (i.e., sperm moving faster than 15 µm s^−1^ on average). Only sperm classified as motile were used for sperm speed analyses.

### Fertilization Success

Data were obtained for 18 pairwise crossings using the same males used in the sperm motility experiments (above). Each male was crossed with one female (no female was spawned for the first male, hence n = 18). Each individual was used only once (18 males and 18 females in total). For each of the three CO_2_ treatments, nine four-fold serially diluted sperm-concentrations and one control (FSW only) were prepared in 6-well plates holding 6 ml of seawater. Sperm concentrations and CO_2_ treatments were distributed across multiple 6-well plates to preclude plate effects. Approximately 200 eggs were added to each well in filter dishes (25 mm diameter × 20 mm height, 80 µm mesh floor). Eggs and sperm were mixed and left for 10 min to fertilize. Eggs were then rinsed twice (with water of the relevant pH) to remove sperm and left for 2 h to develop, (typically this was to the 4 cell stage). Fertilization success rates (FSR) were determined by counting the proportion of cleaved eggs *in vivo*. Sperm concentrations in the stock solutions were checked *post-hoc* using a haemocytometer (Neubauer improved). All fertilizations were conducted within 15 min of obtaining the gametes.

### Modelling of Fertilization Kinetics

**Figure 1:**
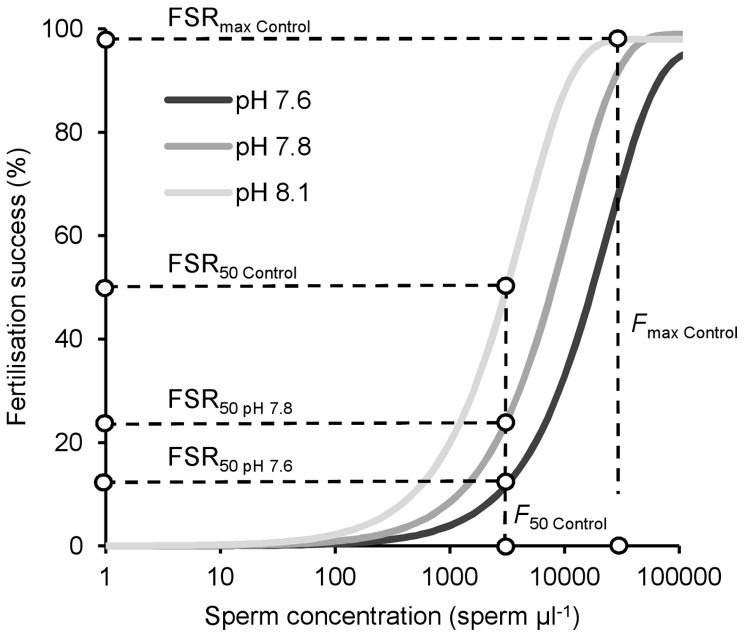
Schematic representation of the relationship between fertilization success and sperm concentration of *Heliocidaris erythrogramma* at different pH levels, assuming a negative pH impact on fertilization. Determining fertilization success at an intermediate sperm concentration (here, the sperm concentration that generates 50% of maximum observed fertilization success in controls) yields maximum sensitivity in the assay. All data in Fig. 1Figure 1 are theoretical. FSR_max Control_ = maximum fertilization success in Controls; FSR_50 Control_ = 50% of maximum fertilization success in Controls; *F*_max Control_ = sperm concentration that generates maximum fertilization success in Controls; *F*_50 Control_ = sperm concentration that generates 50% of maximum fertilization success in Controls. FSR_50 pH 7.8_ = observed fertilization success in pH 7.8 treatment at the sperm concentration that generates 50% of maximum fertilization success in Controls; FSR_50 pH 7.6_ = observed fertilization success in pH 7.6 at the sperm concentration that generates 50% of maximum fertilization success in Controls. Actual fertilization curves vary for each individual pair.

We used modelling [19], [25] to combine the results from motility and fertilization experiments to investigate the importance of pH-induced changes in sperm motility in the fertilization process. Fertilization kinetics curves [20], [26] were fitted to the measured fertilization data for each cross and CO_2_ treatment. Fertilization efficiency (*F_e_*) and polyspermy block efficiency (*B_e_*) were estimated using least squares [27]. Sperm speeds and percentage of motile sperm for each male were taken from video analyses. Sperm concentration for modelling was defined as the product of the observed sperm concentration in the serial dilution and observed percent motility of that male in that treatment. For the control assay (pH 8.1) we identified the sperm concentration that yielded maximum fertilization success (*F*_max Control_; Figure 1), the sperm concentration that yielded 50% of *F*_max_ (*F*_50 Control_ [28]) and the respective fertilization success rates at each of these sperm concentrations (“FSR_max Control_” and “FSR_50 Control_”). For each of the CO_2_ treatments fertilization success at *F*_50 Control_ was observed (“FSR_obs_”) for each cross. These values were then compared to the fertilization success at *F*_50 Control_ that was obtained from the model using the sperm speed and percent motility values observed for each treatment (“FSR_mod_”).

### Data Analyses

All percentage data were arcsine transformed prior to statistical analyses and checked for normality [29]. Levene’s test was used to assess for homogeneity of variances among individuals and among treatments. Two-factor Analysis of Variance (ANOVA) was used to assess pH effects on sperm speed and percent motility across all males (pH fixed, male random). Tukey’s test was used to compare *post-hoc* differences among means. The magnitude of responses of percent motility and fertilization success to pH treatments was assessed using logarithmic responses ratios (LnRR; natural log of treatment response divided by control response [30]). Mean LnRRs and 95% confidence intervals of fertilization success were determined by bootstrapping in R (100,000 iterations; [31]). Regression analysis was used to assess relationships between observed and modelled fertilization outcomes. All statistical tests were carried out using SPSS™.

## Results

### Sperm Motility

We analysed over 141,000 sperm tracks (pH 8.1 n = 43,271; pH 7.8 n = 50,135; pH 7.6 n = 48,330).

**Figure 2:**
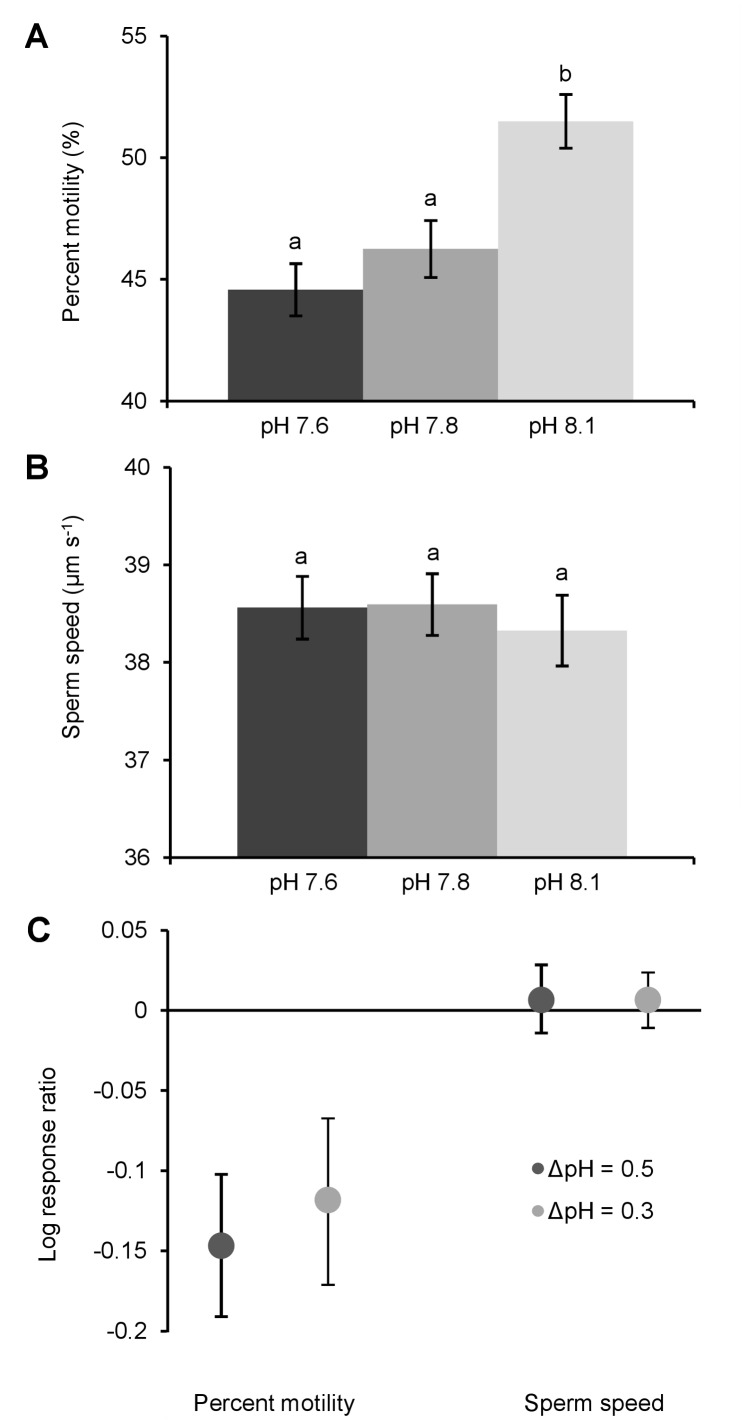
Impacts of ocean acidification on sperm motility and sperm swimming speed in *Heliocidaris erythrogramma.* Proportion of mean (±S.E.) motile sperm (A) and sperm speed (B) at different levels of ocean acidification (pH mediated by CO_2_ addition). Lower case letters indicate significantly different groups at *p* = 0.05 (Tukey’s test). (C) Mean logarithmic response ratios (±95% CI) of effects of ocean acidification on percent motility and sperm speed (n = 19).

Acidification significantly decreased the average percentage of motile sperm (by 7% at ΔpH = 0.3 and 9% at ΔpH = 0.5 pH, *P*<0.001; Fig. 2A, Table 2&3A). Responses of individual males differed significantly (*P*<0.001; Table 3A) with reductions in percentage of motile sperm ranging from −6.5% to −15.7% at ΔpH = 0.3, and −9.7% to −17.4% at ΔpH = 0.5 (Fig. 2C).

**Table 3:** Two-way ANOVA for percent motility (A) and sperm speed (B) of *Heliocidaris erythrogramma* across different pH treatments (fixed) and males (random).

A Percent motility				
	df	MS	F	*P*
pH	2	0.270	24.666	**<0.001**
Male	18	0.531	48.504	**<0.001**
pH * Male	36	0.011	1.226	0.176
Residual	509	0.009		
**B Sperm speed**				
	**df**	**MS**	**F**	***P***
pH	2	4.067	0.346	0.710
Male	18	218.547	18.617	**<0.001**
pH * Male	36	11.739	0.801	0.791
Residual	509	14.654		

Significant effects (*P*≤0.05) are shown in bold.

In contrast, average sperm speed was not significantly affected by acidification (*p* = 0.710; Fig. 2B, Table 2&3B), although again there were significant differences in responses between individuals (*P*<0.001; Table 3B). Upper and lower bound 95% CIs for individual response ratios (LnRRs) of sperm speed were equivalent to +2.4% to −1.1% (ΔpH = 0.3), and +2.9% to −1.4% (ΔpH = 0.5; Fig. 2C). There were no significant differences in sperm parameters between males from different sites at any pH level (Table 2).

### Fertilization Success

**Figure 3:**
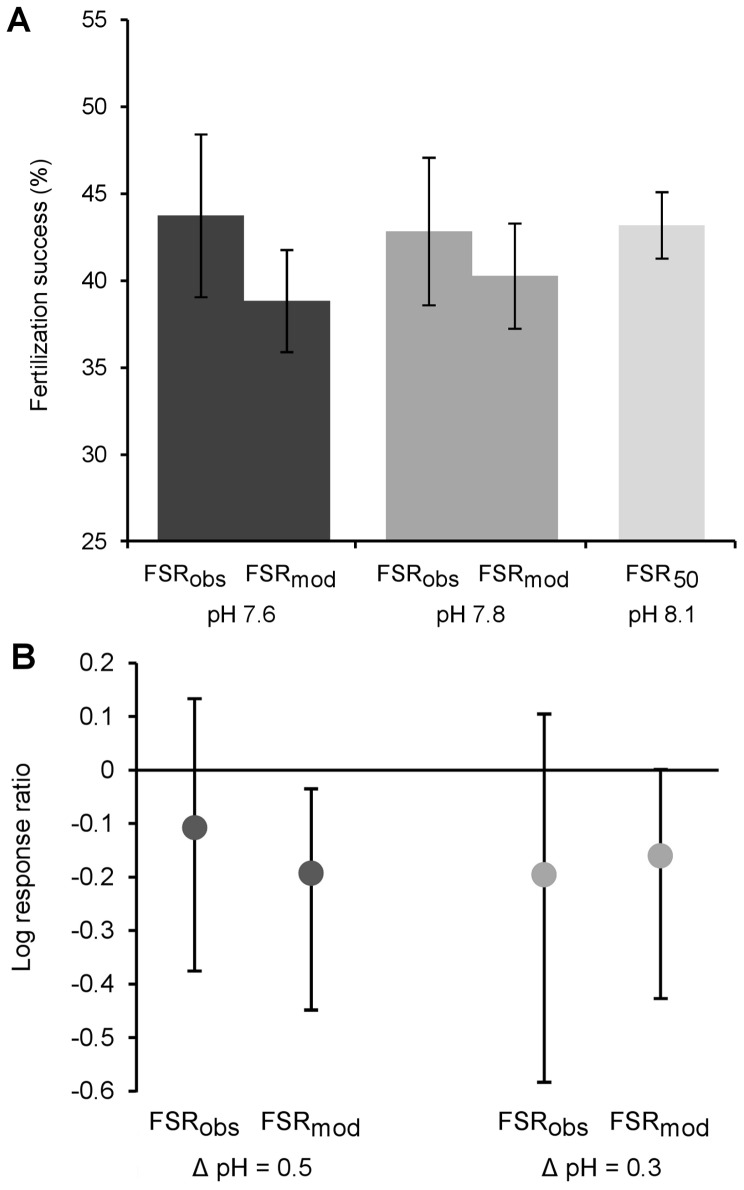
Effects of ocean acidification on fertilization success (FSR) in *H. erythrogramma*. (A) Mean (±S.E.) observed (FSR_obs_) and modelled fertilization success (FSR_mod_) for pHs 7.6 and 7.8, and mean (±S.E.) FSR_50_ (50% of maximum FSR) for pH 8.1. (B) Bootstrapped mean logarithmic response ratios (±95% CI) of effects of ocean acidification on FSR_obs_ and FSR_mod_. FSR_mod_ shows change in fertilization success expected due to ocean acidification’s influence on sperm swimming behaviour (Fig. 2C). (n = 18 replicate trials). See text for details.

Ocean acidification substantially increased the variance of observed (FSR_obs_ in Fig. 3A) and modelled fertilization success (FSR_mod_ in Fig. 3A).

The overall effect of ΔpH on observed fertilization success (Table 4) was not statistically significant (*P = *0.9, Table 5A), however bootstrapping showed that responses were highly variable between pairwise crosses (Fig. 3B, Table 4). The 95% CIs around the mean log response ratios (LnRR) varied from moderately positive (11% and 14% increases in fertilization success at ΔpH = 0.3 and ΔpH = 0.5, respectively) to strongly negative (≤44% and ≤79% decreases at ΔpH = 0.3 and 0.5, respectively; Fig. 3B). There was no significant difference in maximum fertilization success between pairs from different sites (*P* = 0.202).

**Table 4:** Modelled (FSR_mod_) and observed (FSR_obs_) fertilization success for each urchin pair under acidified conditions (pH 7.6 and 7.8), and parameters from Control observations (pH 8.1) used in modelling FSR_mod_ at lowered pH levels.

pH	7.6		7.8		8.1	
Pair/Male	FSR_mod_ (%)	FSR_obs_ (%)	FSR_mod_ (%)	FSR_obs_ (%)	FSR_50 Control_ (%)	*F*_50 Control_ (sperm µl^−1^)
A	no data					
B	26.46	63.92	25.33	50.38	28.95	1258.93
C	36.21	47.93	55.81	47.19	47.76	1584.89
D	23.25	10.46	24.45	12.95	24.94	4466.84
E	41.34	45.80	43.21	62.59	37.03	3162.28
F	51.59	24.21	50.45	37.27	49.74	7943.28
G	41.55	44.83	42.74	46.93	48.98	2511.89
H	38.78	61.78	39.09	51.80	47.37	22387.21
I	41.52	9.05	43.45	7.11	42.03	251.19
J	3.74	30.15	3.50	2.47	31.94	398.11
K	38.95	71.08	41.06	53.18	48.64	22387.21
L	40.78	64.17	41.26	60.09	48.06	10000.00
M	28.60	40.79	30.54	35.49	34.61	2511.89
N	52.12	47.55	53.29	53.32	48.81	3981.07
O	35.89	43.26	37.83	43.39	42.95	10000.00
P	43.72	28.96	41.66	52.20	49.88	50118.72
Q	49.49	22.29	49.71	41.97	48.26	8912.51
R	50.57	54.19	47.54	63.00	46.58	15848.93
S	54.44	76.84	53.87	49.54	50.77	2238.72
**mean**	38.83	43.74	40.27	42.83	43.18	9442.43
**S.E.**	2.93	4.68	3.02	4.24	1.91	2898.53

FSR_50 Control_ = 50% of maximum fertilization success in Controls; *F*_50 Control_ = sperm concentration that generates 50% of maximum fertilization success in Controls. Sperm data from each male in Table 2 were used in modelling FSR_mod_. No females were spawned for male A.

**Table 5:** Two-way ANOVA for observed (FSR_obs_; A) and modelled fertilization success (FSR_mod_; B) of *Heliocidaris erythrogramma* across different pH treatments (fixed) and males (random).

A FSR_obs_				
	df	MS	F	*P*
pH	2	0.002	0.103	0.902
Male	17	0.065	3.711	**0.001**
pH * Male	34	0.018	18.794	0.181
Residual	1	0.001		
**B FSR_mod_**				
	**df**	**MS**	**F**	***P***
pH	2	0.013	3.097	0.058
Male	17	0.045	11.005	**<0.001**
pH * Male	34	0.004	4.308	0.367
Residual	1	0.001		

Significant effects (P≤0.05) are shown in bold.

**Figure 4:**
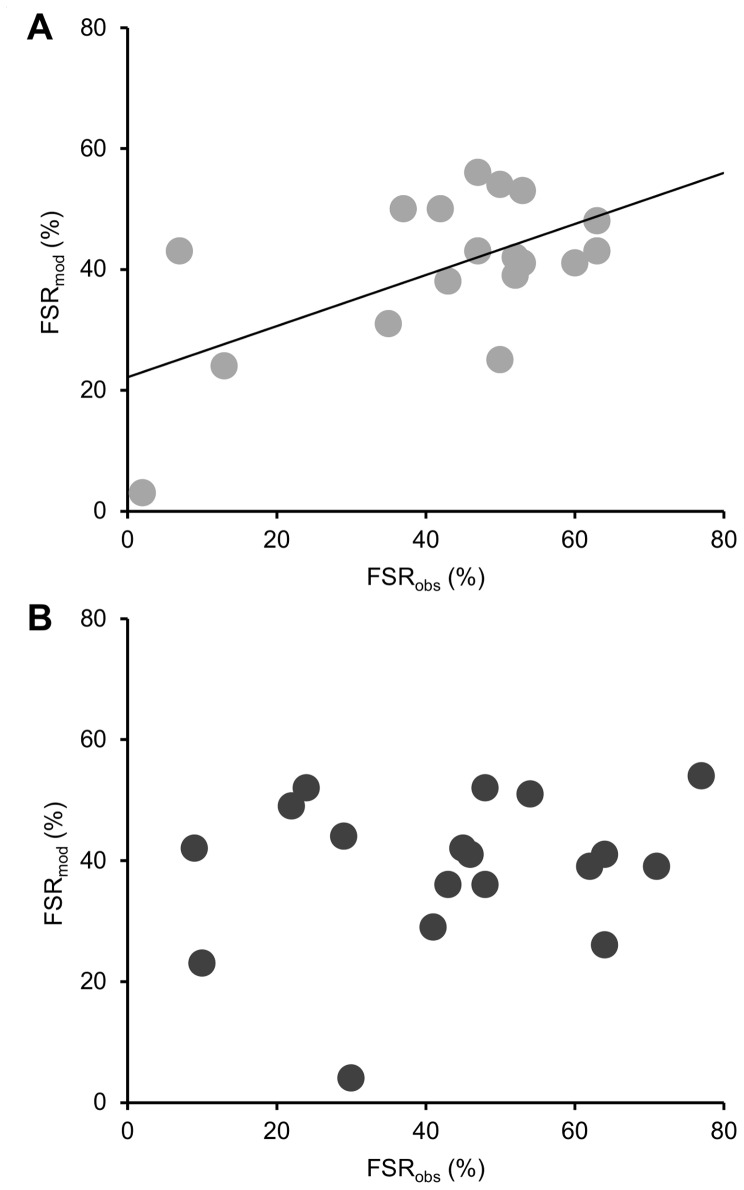
Scatterplots for observed (FSR_obs_) versus modelled (FSR_mod_) fertilization success for pH 7.8 (A) and 7.6 (B). Regression analyses revealed a significant relationship between observed (independent) and modelled fertilization (dependent) for pH 7.8 (*P* = 0.012, r^2^ = 0.336), but not for pH 7.6 (*P* = 0.413, r^2^ = 0.042).

Modelling the effects of the observed changes in sperm percent motility and sperm swimming speeds on fertilization success yielded predictions that broadly mirrored the patterns seen in observed measurements (FSR_mod_ in Fig. 3B, Table 5B): for ΔpH = 0.3 the modelled LnRR 95% CI ranged from an equivalent of 0 to −34.8%, and for ΔpH = 0.5 from −3.4% to −36.1%. Regression analyses (Fig. 4) revealed that 34% of the observed change in fertilization success could be attributed to changes in sperm motility at ΔpH = 0.3, but only 4% at ΔpH = 0.5.

## Discussion

Our finding that the effects of acidification on sperm swimming behaviour (Fig. 2C, Table 2), and fertilization success (Fig. 3, Table 4), vary significantly between individuals is biologically important. Differences in individual responses are the raw material for effective selection, and especially so at the critical life stage of fertilization [2]. However, inter-individual variation has been overlooked in most previous, group-mean based investigations.

The shortcomings of analysing group means are demonstrated by comparing the overall non-significant effect of ocean acidification on mean fertilization (Table 5) with the substantial inter-individual variation in response we observed (FSR_obs_ in Fig. 3B). The majority of individual pairs had reduced fertilization success under acidified conditions, however, some pairs showed increased fertilization success (Fig. 3B, Table 4). This illustrates the importance of examining individual responses: it is individuals that contribute differentially to the next generation – not group means. Consequently, approaches that assess the group mean response ignore evolutionarily important effects of rare individuals that may contribute disproportionately to the next generation. This example also emphasizes the need for adequate sample sizes in order to capture the variety of individual responses to ocean acidification, particularly in species with high inter-individual variation.

The effects of acidification on fertilization success have often been tested using inappropriately high sperm concentrations. Using a sperm concentration that yields maximum fertilization success in controls (*F*_max_ in Fig. 1) can generate misleading or uninformative results, because assays may be saturated and therefore unresponsive to subtle, but biologically important, differences in fertilization [32], and because such assays cannot detect possible positive effects of the treatment. Had such high concentrations been used in our experiments, the observed increases in fertilization success seen in some pairs would not have been detected (Fig. 3). Choosing 50% of the maximum fertilization success as the response variable (*F*_50_ in Fig. 1; [28]) allowed us to detect both negative and positive pH impacts on fertilization success, while ensuring maximum sensitivity in our assays.

Previous investigations of the effects of ocean acidification on fertilization success in *H. erythrogramma* have found contradictory results [11], [33], [34]. These differences may partly be explained by the use of saturated assays in some studies. Previous studies also used smaller sample sizes than used here (5 M × F pairs [11], 3 replicates [33], [34], *vs* 18 M × F pairs in this study). The use of gametes mixed from multiple individuals [33], [34] also precludes observation of intra-specific variation. Apparently conflicting results may also be explained by the intra-specific variation demonstrated in our experiments. Fertilization success in some pairs was negatively impacted by acidification (confirming [11]) whereas other pairs showed no (or little) net response (confirming [33], [34]). Thus, we suggest that much of the controversy around the response of fertilization success in *H. erythrogramma* to ocean acidification can be attributed to a combination of the factors discussed above.

Our observation that lowered seawater pH did not affect the speed of motile sperm (Fig. 2B) but rather decreased the proportion of motile sperm (Fig. 2A) contrasts partly with earlier reports [11]. Sea urchin sperm are stored immotile in an acidic environment inside the testis after development, which inhibits respiration and metabolic processes prior to release [35]–[39]. Upon spawning, the difference between intra-cellular pH in the testis and extracellular pH in the seawater triggers mitochondrial activity and thus motility. Ocean acidification may reduce the pH gradient upon spawning to a point where it is insufficient to activate the sperm mitochondrion. Since we observed a decrease in the proportion of motile sperm under acidified conditions (Fig. 2A), but the swimming speed of those motile sperm did not decrease (Fig. 2B), this strongly suggests an effect on activation of the sperm mitochondrion but not on mitochondrial function once activated. As sperm have no actively transcribing nuclear genes or biochemistry, the most parsimonious explanation for our results is that genetic variation in mitochondrial membrane protein genes explains some of the observed inter-individual variation in sperm swimming behaviour, and hence fertilization success.

Variation in fertilization success may also have been influenced by parental environmental history, although our results suggest this was not likely to have been a significant driving factor. Individuals were collected from small areas of uniform habitat, and variance within single populations was as large as differences between populations (the converse would be expected if environmental history effects were more influential than genetic diversity). Early cleavage stages (2 h post-fertilization) are also largely independent of transcription of paternal DNA [40]. Consequently we suggest that variation in sperm swimming behaviour and sperm-egg binding compatibility [41], [42] are the most likely explanations for the observed variance in responses to acidification.

More broadly, for free spawners such as *H. erythrogramma* the chance of successful fertilization depends on gamete concentrations, gamete life span and (hence) on the distance between spawning individuals [14], [43]. The density and abundance of *H. erythrogramma* varies substantially *in situ* – from <1 to >50 individuals.m^−2^ [44], [45], occurring in a range of even to patchy distributions. Low fertilization success due to sperm limitation may be common between widespread clusters of urchins because sperm longevity is short [46]–[48]. If many individuals within populations display decreased sperm motility due to ocean acidification, sperm limitation downstream of a spawning site may be exacerbated [49], [50], further decreasing reproductive success and ultimately reducing the number of individuals that contribute to future generations [15], [51].

Our results support the concept of ‘winners’ and ‘losers’ of climate change; a concept often proposed over the last decade [52]–[55], and applied to many organisational levels such as alleles within genes, individuals within populations, and species within ecosystems. Here we apply the ‘winners’ and ‘losers’ concept at the individual level. The substantial variation in sperm motility and fertilization success we observed in response to predicted ocean acidification (Figs 2, 3) shows that some individuals (here, males or male x female pairs, see above) are better equipped than others to cope with acidification. Future ocean acidification will likely reduce the proportion of fertilizations by acidification-sensitive gametes (‘losers’), and increase the proportion of fertilizations by acidification-resistant gametes (‘winners’). Whether these ‘winners’ are selected due to genetic traits (such as sperm swimming ability (Fig. 2A) and sperm-egg binding compatibilities [41], [42]) or due to non-genetic maternal traits (such as egg condition [56]) will be immaterial to the selection of the ‘winners’ *per se*. It also remains unclear whether ‘winners’ of climate change-induced selection for fertilization success will actually remain ‘winners’ across the entire life-cycle. Nonetheless, it should be remembered that the heritability of the selected traits will of course strongly influence the future adaptation potential of the progeny and hence the long-term adaptive benefits of ‘winner’ status.

If this observed variability is heritable, it will have important implications for urchin populations in a near-future acidified ocean. Loss of less competitive gamete genotypes will reduce overall genetic diversity, at least initially. This loss may be counteracted by genetic drift and new mutations, although in the short term these are likely to be trivial compared to the effects of selection. The long-term fitness consequences, however, will depend on the fitness benefits of traits that covary with acidification-resistance in gametes, and the extent to which recombination in second and subsequent generations gather advantageous alleles in some individuals. Any selection that reduces available genetic diversity leaves future populations less capable of tolerating further perturbations [57], [58], and this underscores the need for analyses of the fitness of the descendants resulting from experiments such as those we have described here.

In summary, despite an increasing number of studies focusing on the effects of ocean acidification on the early life history of marine organisms [59], [60], very few studies have investigated individual-level responses to changing oceanic conditions. This is perplexing in light of the growing evidence for a degree of inter-individual variation that exceeds the noise of baseline variability [24]. Conducting adequately replicated studies to investigate inter-individual variability in response to marine climate change is imperative if we are to understand the capacity for selection and adaptation of marine species.

## References

[1] Levitan DR (1996) Effects of gamete traits on fertilization in the sea and the evolution of sexual dimorphism. Nature 382: 153–155.

[2] Levitan DR (2008) Gamete traits influence the variance in reproductive success, the intensity of sexual selection, and the outcome of sexual conflict among congeneric sea urchins. Evolution 62: 1305–1316. 18363865 10.1111/j.1558-5646.2008.00378.x

[3] IPCC (2007) The 4th Assessment Report of the IPCC. Cambridge, UK.

[4] Doney SC, Fabry VJ, Feely RA, Kleypas JA (2009) Ocean Acidification: The Other CO_2_ Problem. Annu Rev Mar Sci 1: 169–192. 10.1146/annurev.marine.010908.163834 21141034

[5] Kroeker KJ, Kordas RL, Crim RN, Singh GG (2010) Meta-analysis reveals negative yet variable effects of ocean acidification on marine organisms. Ecol Lett 13: 1419–1434. 20958904 10.1111/j.1461-0248.2010.01518.x

[6] Doney SC, Ruckelshaus M, Duffy JE, Barry JP, Chan F, et al (2012) Climate Change Impacts on Marine Ecosystems. Annu Rev Mar Sci 4: 4.1–4.27. 10.1146/annurev-marine-041911-111611 22457967

[7] Doo SS, Dworjanyn SA, Foo SA, Soars NA, Byrne M (2011) Impacts of ocean acidification on development of the meroplanktonic larval stage of the sea urchin *Centrostephanus rodgersii*. ICES J Mar Sci.

[8] Dupont S, Havenhand J, Thorndyke W, Peck L, Thorndyke M (2008) Near-future level of CO_2_-driven ocean acidification radically affects larval survival and development in the brittlestar *Ophiothrix fragilis* . Mar Ecol Prog Ser 373: 285–294.

[9] Martin S, Richier S, Pedrotti M-L, Dupont S, Castejon C, et al (2011) Early development and molecular plasticity in the Mediterranean sea urchin *Paracentrotus lividus* exposed to CO_2_-driven acidification. J Exp Biol 214: 1357–1368. 21430213 10.1242/jeb.051169

[10] O'Donnell MJ, Todgham AE, Sewell MA, Hammond LM, Ruggiero K, et al (2010) Ocean acidification alters skeletogenesis and gene expression in larval sea urchins. Mar Ecol Prog Ser 398: 157–171.

[11] Havenhand JN, Buttler FR, Thorndyke MC, Williamson JE (2008) Near-future levels of ocean acidification reduce fertilization success in a sea urchin. Curr Biol 18: R651–R652. 18682203 10.1016/j.cub.2008.06.015

[12] Sunday JM, Crim RN, Harley CDG, Hart MW (2011) Quantifying Rates of Evolutionary Adaptation in Response to Ocean Acidification. PLoS ONE 6: e22881. 21857962 10.1371/journal.pone.0022881 PMC3153472

[13] Chan KYK, Grünbaum D, O'Donnell MJ (2011) Effects of ocean-acidification-induced morphological changes on larval swimming and feeding. J Exp Biol 214: 3857–3867. 22031751 10.1242/jeb.054809

[14] Levitan DR, Sewell MA, Chia FS (1992) How distribution and abundance influence fertilization success in the sea-urchin *Strongylocentrotus franciscanus* . Ecology 73: 248–254.

[15] Reuter KE, Lotterhos KE, Crim RN, Thompson CA, Harley CDG (2011) Elevated pCO_2_ increases sperm limitation and risk of polyspermy in the red sea urchin *Strongylocentrotus franciscanus* . Glob Change Biol 17: 163–171.

[16] Andrew NL (1993) Spatial heterogeneity, sea-urching grazing, and habitat structure on reefs in temperate Australia. Ecology 74: 292–302.

[17] Harrold C, Reed DC (1985) Food availability, sea urchin grazing, and kelp forest community structure. Ecology 66: 1160–1169.

[18] Evans JP, Marshall DJ (2005) Male-by-female interactions influence fertilization success and mediate the benefits of polyandry in the sea urchin *Heliocidaris erythrogramma* . Evolution 59: 106–112. 15792231

[19] Vogel H, Czihak G, Chang P, Wolf W (1982) Fertilization kinetics of sea urchin eggs. Math Biosci 58: 189–216.

[20] Styan CA, Kupriyanova E, Havenhand JN (2008) Barriers to cross-fertilization between populations of a widely dispersed polychaete species are unlikely to have arisen through gametic compatibility arms-races. Evolution 62: 3041–3055. 18803690 10.1111/j.1558-5646.2008.00521.x

[21] Dickson AG (2010) The carbon dioxide system in seawater: equilibrium chemistry and measurements. In: Riebesell U, Fabry VJ, Hansson L, Gattuso J-P, editors. Guide to best practices for ocean acidification research and data reporting. Luxembourg: Publications Office of the European Union. 17–40.

[22] Lewis E, Wallace D (1998) Program developed for CO_2_ system calculations. Oak Ridge, Tennessee: ORNL/CDIAC-105: Carbon Dioxide Information Analysis Center, Oak Ridge National Laboratory, U.S. Department of Energy.

[23] Dickson AG, Millero FJ (1987) A comparison of the equilibrium constants for the dissociation of carbonic acid in seawater media. Deep Sea Res Pt A 34: 1733–1743.

[24] Havenhand JN, Schlegel P (2009) Near-future levels of ocean acidification do not affect sperm motility and fertilization kinetics in the oyster *Crassostrea gigas* . Biogeosciences 6: 3009–3015.

[25] Styan CA (1998) Polyspermy, egg size, and the fertilization kinetics of free-spawning marine invertebrates. Am Nat 152: 290–297. 18811392 10.1086/286168

[26] Styan CA, Byrne M, Franke E (2005) Evolution of egg size and fertilisation efficiency in sea stars: large eggs are not fertilised more readily than small eggs in the genus *Patiriella* (Echinodermata: Asteroidea). Mar Biol 147: 235–242.

[27] Styan CA, Butler AJ (2000) Fitting fertilisation kinetics models for free-spawning marine invertebrates. Mar Biol 137: 943–951.

[28] Levitan DR (2002) The Relationship between Conspecific Fertilization Success and Reproductive Isolation among Three Congeneric Sea Urchins. Evolution 56: 1599–1609. 12353753 10.1111/j.0014-3820.2002.tb01472.x

[29] Quinn G, Keough M (2002) Experimental design and data analysis for biologists: Cambridge University Press.

[30] Nakagawa S, Cuthill IC (2007) Effect size, confidence interval and statistical significance: a practical guide for biologists. Biol Rev 82: 591–605. 17944619 10.1111/j.1469-185X.2007.00027.x

[31] R-Development-Core-Team (2012) R: A language and environment for statistical computing. Vienna, Austria: R Foundation for Statistical Computing.

[32] Marshall DJ (2006) Reliably estimating the effect of toxicants on fertilization success in marine broadcast spawners. Mar Pollut Bull 52: 734–738. 16797034 10.1016/j.marpolbul.2006.05.005

[33] Byrne M, Ho M, Selvakumaraswamy P, Nguyen HD, Dworjanyn SA, et al (2009) Temperature, but not pH, compromises sea urchin fertilization and early development under near-future climate change scenarios. Proc R Soc B-Biol Sci 276: 1883–1888. 10.1098/rspb.2008.1935 PMC2674501 19324767

[34] Byrne M, Soars N, Selvakumaraswamy P, Dworjanyn SA, Davis AR (2010) Sea urchin fertilization in a warm, acidified and high pCO_2_ ocean across a range of sperm densities. Mar Environ Res 69: 234–239. 19913293 10.1016/j.marenvres.2009.10.014

[35] Christen R, Schackmann RW, Shapiro BM (1983) Metabolism of Sea Urchin Sperm: Interrelationships between intracellular pH, ATPase activity, and mitochondrial respiration. J Biol Chem 258: 5392–5399. 6222053

[36] Christen R, Schackmann RW, Shapiro BM (1982) Elevation of the intracellular pH activates respiration and motility of sperm of the sea urchin, *Strongylocentrotus purpuratus* . J Biol Chem 257: 14881–14890. 7174670

[37] Christen R, Schackmann RW, Dahlquist FW, Shapiro BM (1983) ^31^P-NMR analysis of sea urchin sperm activation: Reversible formation of high energy phosphate compounds by changes in intracellular pH. Exp Cell Res 149: 289–294. 6641799 10.1016/0014-4827(83)90400-7

[38] Hamamah S, Gatti J-L (1998) Role of the ionic environment and internal pH on sperm activity. Hum Reprod 13: 20–30. 10.1093/humrep/13.suppl_4.20 10091055

[39] Lee HC, Johnson CR, Epel D (1983) Changes in internal pH associated with initiation of motility and acrosome reaction of sea urchin sperm. Dev Biol 95: 31–45. 6825930 10.1016/0012-1606(83)90004-0

[40] Tadros W, Lipshitz HD (2009) The maternal-to-zygotic transition: a play in two acts. Development 136: 3033–3042. 19700615 10.1242/dev.033183

[41] Palumbi SR (1999) All males are not created equal: Fertility differences depend on gamete recognition polymorphisms in sea urchins. Proc Nat Acad Sci 96: 12632–12637. 10535974 10.1073/pnas.96.22.12632 PMC23023

[42] Levitan DR, Ferrell DL (2006) Selection on gamete recognition proteins depends on sex, density, and genotype frequency. Science 312: 267–269. 16614223 10.1126/science.1122183

[43] Levitan DR (2002) Density-dependent selection on gamete traits in three congeneric sea urchins. Ecology 83: 464–479.

[44] Wright JT, Steinberg PD (2001) Effect of variable recruitment and post-recruitment herbivory on local abundance of a marine alga. Ecology 82: 2200–2215.

[45] Wright JT, Dworjanyn SA, Rogers CN, Steinberg PD, Williamson JE, et al (2005) Density-dependent sea urchin grazing: differential removal of species, changes in community composition and alternative community states. Mar Ecol Prog Ser 298: 143–156.

[46] Pennington JT (1985) The ecology of fertilization of echinoid eggs: the consequences of sperm dilution, adult aggregation, and synchronous spawning. Biol Bull 169: 417–430. 29314924 10.2307/1541492

[47] Levitan DR (2000) Sperm velocity and longevity trade off each other and influence fertilization in the sea urchin *Lytechinus variegatus* . Proc R Soc Lond Ser B-Biol Sci 267: 531–534. 10.1098/rspb.2000.1032 PMC1690568 10787153

[48] Levitan DR, Petersen C (1995) Sperm limitation in the sea. Trends Ecol Evol 10: 228–231. 21237018 10.1016/S0169-5347(00)89071-0

[49] Babcock RC, Mundy CN, Whitehead D (1994) Sperm Diffusion Models and *In Situ* Confirmation of Long-Distance Fertilization in the Free-Spawning Asteroid *Acanthaster planci* . Biol Bull 186: 17–28. 29283304 10.2307/1542033

[50] Levitan DR (2004) Density-dependent sexual selection in external fertilizers: Variances in male and female fertilization success along the continuum from sperm limitation to sexual conflict in the sea urchin *Strongylocentrotus franciscanus* . Am Nat 164: 298–309. 15478086 10.1086/423150

[51] Levitan DR, Young CM (1995) Reproductive success in large populations: empirical measures and theoretical predictions of fertilization in the sea biscuit *Clypeaster rosaceus* . J Exp Mar Biol Ecol 190: 221–241.

[52] O'Brien KL, Leichenko RM (2000) Double exposure: assessing the impacts of climate change within the context of economic globalization. Glob Environ Change 10: 221–232.

[53] Loya Y, Sakai K, Yamazato K, Nakano Y, Sambali H, et al (2001) Coral bleaching: the winners and the losers. Ecol Lett 4: 122–131.

[54] Somero GN (2010) The physiology of climate change: how potentials for acclimatization and genetic adaptation will determine ‘winners’ and ‘losers’. J Exp Biol 213: 912–920. 20190116 10.1242/jeb.037473

[55] Glantz MH (1995) Assessing the impacts of climate: The issue of winners and losers in a global climate change context. Stud Environ Sci 65: 41–54.

[56] Byrne M, Prowse TAA, Sewell MA, Dworjanyn S, Williamson JE, et al (2008) Maternal provisioning for larvae and larval provisioning for juveniles in the toxopneustid sea urchin *Tripneustes gratilla* . Mar Biol 155: 473–482.

[57] Reed DH, Frankham R (2003) Correlation between Fitness and Genetic Diversity. Conserv Biol 17: 230–237.

[58] Frankham R (2005) Conservation Biology: Ecosystem recovery enhanced by genotypic diversity. Heredity 95: 183–183. 16049423 10.1038/sj.hdy.6800706

[59] Kurihara H (2008) Effects of CO_2_-driven ocean acidification on the early developmental stages of invertebrates. Mar Ecol Prog Ser 373: 275–284.

[60] Byrne M (2011) Impact of ocean warming and ocean acidification on marine invertebrate life history stages : Vulnerabilities and potential for persistence in a changing ocean. Oceanogr Mar Biol 49: 1–42.

